# Supplementary material for: Opportunities and Challenges to Improve a Public Research Program in Plant Breeding and Enhance Underutilized Plant Genetic Resources in the Tropics
Source: Genes (Basel). 2021 Oct 9;12(10):1584. doi: 10.3390/genes12101584 (PMC8535561; doi:10.3390/genes12101584)
Supplement: Supplementary file 1 [file genes-12-01584-s001.zip › Supplementary Materials File S1.pdf]

Artículo

Oportunidades y desafíos para mejorar un programa público de investigación en fitomejoramiento y mejorar los recursos fitogenéticos subutilizados en los trópicos

Ivania Cerón-Souza[1,5]*, Carlos H. Galeano[2,3], Katherine Tehelen[4], Hugo R. Jiménez[1] y Carolina González[1]

[1] Corporación Colombiana de Investigación Agropecuaria – AGROSAVIA, C.I. Tibaitatá, Mosquera, Colombia.; iceron@agrosavia.co, hjimenez@agrosavia.co, cgonzaleza@agrosavia.co.
[2] Corporación Colombiana de Investigación Agropecuaria – AGROSAVIA, C.I. Palmira, Palmira, Colombia.
[3] Dirección actual: BASF Vegetable Seeds, Davis, CA, U.S.A.; cgaleano@gmail.com.
[4] Consultant in Environmental Economy, Davis, CA, U.S.A..; katherine.tehelen@gmail.com.
[5] Estos autores contribuyeron por igual: Ivania Cerón-Souza y Carlos H. Galeano.
* Correspondencia: iceron@agrosavia.co; Tel.: +57144227300 ext. 1457

Resumen

Los trópicos americanos son focos de biodiversidad de plantas silvestres y domesticadas, gran parte de la cual sigue siendo subutilizada por los programas de mejora genética a pesar de estar conservada en bancos de genes regionales. La mejora de esos programas depende de fondos públicos a largo plazo y del mantenimiento de personal especializado. Por desgracia, los altibajos financieros complican la conectividad del personal y su impacto en la investigación. Entre 2000 y 2010, AGROSAVIA (Corporación Colombiana de Investigación Agropecuaria) disminuyó drásticamente su apoyo financiero público. En 2017, encuestamos a los 52 investigadores de AGROSAVIA involucrados en programas de mejoramiento vegetal y recursos fitogenéticos para examinar el impacto de la disminución de fondos en los últimos diez años. Nuestra hipótesis es que el personal dedicado a la mejora vegetal sigue sufriendo una fuerte fragmentación y una baja conectividad. Como esperábamos, la red social entre los investigadores es débil. Los diez principales líderes centrales son predominantemente hombres con un título de maestría, pero tienen una experiencia significativa en el área. El personal tiene experiencia en 31 cultivos tropicales, y 17 están en la lista de especies infrautilizadas. Además, aunque 26 de estos cultivos están en el banco nacional de germoplasma, éste no ha sido la fuente principal de sus programas de mejoramiento. Propusimos cinco principios para mejorar la conectividad entre equipos y el impacto de la investigación. Estos principios también podrían aplicarse en otros países tropicales con programas públicos de investigación en fitomejoramiento que se enfrentan a retos similares.

Palabras clave: agrobiodiversidad; seguridad alimentaria; brecha de género; bancos de germoplasma; redes sociales.

1. Introducción

La mayoría de los puntos calientes de biodiversidad del mundo se encuentran en los trópicos. Además, esta ecozona se solapa con el 28% de los centros de domesticación de cultivos. De estos centros de domesticación, América Latina tropical es fundamental. Algunos de los cultivos que se originaron y diversificaron en esta región, como el maíz, la papa y la yuca, son de importancia mundial. Sin embargo, muchos otros son importantes sólo a nivel local, por lo que mantienen una categoría de especies desatendidas y subutilizadas (NUS)[1].

A pesar de estas extraordinarias condiciones, los latinoamericanos se enfrentan a muchos problemas entremezclados, como la pobreza, la inseguridad alimentaria y el cambio climático [2]. Así, el futuro de América Latina es un reto en un contexto en el que la población humana alcanzará los 9.000 millones de personas en 2050 [3]. En estas condiciones alarmantes, la producción mundial de alimentos debe aumentar del 25% al 70% respecto a los niveles de 2014 [3]. En este contexto, los programas de fitomejoramiento en América Latina son críticos, especialmente para los NUS [4-6]. Estas especies son fundamentales para garantizar la productividad de los cultivos (es decir, el rendimiento, la resistencia a enfermedades y plagas), la seguridad alimentaria y de nutrientes, y la adaptación de los cultivos específicos al cambio climático (es decir, la tolerancia al calor, las heladas, la sequía o la salinidad) [7-10]. Además, podrían contribuir a reducir la mortalidad infantil en los hogares pobres [11].

En Colombia, al igual que en muchos países tropicales de América Latina, la inversión en programas de fitomejoramiento y recursos fitogenéticos (PB&PGR) que utilizan la agrobiodiversidad regional ha tenido altibajos debido a una economía a merced del conflicto interno [12,13]. El acuerdo de paz firmado con la guerrilla de las FARC en 2016 reconoció esos problemas históricos. Por ello, incluyó la inversión pública en la mejora de la calidad de vida en las zonas rurales, donde la agricultura es la principal fuente de ingresos, al tiempo que hizo un fuerte hincapié en el uso sostenible de la biodiversidad colombiana [14,15]. Además, como consecuencia de los movimientos agrarios entre 2013-2014, la discusión nacional sobre la agricultura se desplazó hacia la provisión de alimentos en Colombia en un contexto de soberanía [16]. Por lo tanto, los programas públicos de investigación asociados al fitomejoramiento requieren un enfoque multidisciplinario y multisectorial fuerte que el país perdió tras muchos años de falta de inversión y que ahora debe reconstruir [17].

La investigación colombiana en materia de fitomejoramiento comenzó en 1955, dirigida por el DIA (Departamento de Investigaciones Agrarias). Muchos expertos del DIA desarrollaron las primeras variedades vegetales de éxito en diferentes cultivos, como el maíz y la papa (es decir, Diacol H104 y Diacol Capiro, respectivamente). En 1962, el DIA se convirtió en el ICA, que inició una colaboración esencial con la Fundación Rockefeller, potenciando el fitomejoramiento, la fitopatología y la fisiología de las plantas [18]. El ICA también generó becas y subvenciones para apoyar los estudios de maestría y doctorado y la formación de investigadores fuera del país para muchos investigadores. Además de esto, el ICA lideró la creación y consolidación del

Banco Nacional de Germoplasma de Colombia con fines agrícolas y varios programas de mejoramiento genético con un alcance de investigación multidisciplinario [19]. Ambas iniciativas gubernamentales contribuyeron a establecer el primer programa nacional de maestría en genética y el primer programa de mejoramiento genético en colaboración con la Universidad Nacional de Colombia [20].

Como consecuencia de toda esta colaboración e inversión en recursos humanos, el ICA produjo y liberó muchas variedades vegetales durante los siguientes 30 años, entre las décadas de 1960 y 1980, entre las que destacan el maíz, el trigo, la cebada, la avena, el arroz y la papa, que generaron grandes impactos y beneficios para los productores del país [20,21]. A principios de la década de 1990, el gobierno creó AGROSAVIA (antes conocida como Corpoica) como institución nacional de investigación en agricultura, dejando al ICA sólo a cargo de las funciones de control, inspección y vigilancia fitosanitaria. Desafortunadamente, entre 2000-2010, las prioridades del gobierno cambiaron hacia una economía de inversión en la guerra, y el presupuesto de investigación para la investigación agrícola, incluyendo los programas de fitomejoramiento, se vio severamente afectado [17]. Debido a estas decisiones, el objetivo de la investigación nacional en materia de fitomejoramiento pasó a ser la evaluación sobre el terreno de las variedades vegetales desarrolladas por centros internacionales como el CIAT (Centro Internacional de Agricultura Tropical), el CIMMYT (Centro Internacional de Mejoramiento de Maíz y Trigo) y el CIP (Centro Internacional de la Papa).

En 2014, el Congreso de Colombia aprobó el Proyecto de Ley Nº 1731, que prometió asignar un presupuesto anual para apoyar y promover los programas de investigación agrícola en AGROSAVIA. Desde entonces, AGROSAVIA ha renovado progresivamente su capacidad de investigación mediante la incorporación de nuevo personal investigador y nuevas infraestructuras. En la actualidad, el núcleo central de investigadores que trabajan en las áreas de fitomejoramiento y fitogenética son jóvenes investigadores de doctorado formados en metodologías y herramientas convencionales y de vanguardia para seleccionar y desarrollar nuevas variedades vegetales. Sin embargo, AGROSAVIA sigue experimentando con diferentes estrategias para cumplir sus objetivos. De 2014 a 2016, el enfoque principal fue la creación de redes de investigación basadas en las cadenas nacionales de producción agrícola. A partir de 2017, AGROSAVIA ha estado trabajando para consolidar grupos disciplinarios [17]. El objetivo de ambos planes fue renovar el liderazgo en la investigación agrícola utilizando recursos públicos. Por lo tanto, en este estudio, se pretende identificar las brechas significativas en las interacciones entre los investigadores y los retos de investigación para crear una política de reactivación de la investigación en recursos fitogenéticos y mejoramiento vegetal público en Colombia liderada por AGROSAVIA. Entre 2000 y 2010, AGROSAVIA disminuyó su apoyo financiero, creando una competencia interna por los recursos y utilizando el personal de doctorado y maestría principalmente para la asesoría externa [17]. Como consecuencia de ello, planteamos la hipótesis de que el personal dedicado a la mejora genética vegetal y a los recursos fitogenéticos (PB&PGR) sigue sufriendo una fuerte fragmentación y una baja conectividad, lo que conlleva un bajo impacto en esta área que los investigadores son capaces de percibir. Nuestro análisis apoyó esta hipótesis, por lo que proponemos cinco principios que ayudarán a reactivar el liderazgo de la investigación pública en PB&PGR liderada por AGROSAVIA.

2. Materiales y métodos

En febrero de 2017 encuestamos a los 52 investigadores de AGROSAVIA que trabajaban en PB&PGR. Diseñamos un cuestionario online de Google dividido en tres secciones: la descripción del grupo, los retos y oportunidades en el área de PB&PGR, y las interacciones sociales dentro del grupo de PB&PGR y con investigadores externos (Apéndice A). Visualizamos las respuestas utilizando los paquetes R Plotrix 3.7-4 [22] y ggplot2 3.3.1 [23] en R Studio 1.2.5001.

2.1. La descripción del grupo

Comparamos y visualizamos el número de investigadores a través de la edad, sexo, el grado académico más alto, los años de experiencia y la categoría de rango entre los 52 investigadores del grupo PB&PGR en AGROSAVIA.

2.2. Los retos y oportunidades en el área de PB&PGR

2.2.1. Lista de cultivos con interés de investigación y métodos de mejora utilizados

Primero pedimos a los 52 investigadores encuestados que enumeraran todos los cultivos en los que habían trabajado en los últimos diez años. A continuación, preguntamos a los investigadores en qué cultivos querían seguir trabajando. Las listas se compararon y posteriormente se separaron en tres categorías: interés creciente, interés igual o interés decreciente. También se pidió a los investigadores que informaran de los métodos de mejora vegetal utilizados para cada uno de los cultivos enumerados. Utilizamos la lista de cultivos de los investigadores encuestados y la comparamos con tres listas diferentes de información disponible: La lista de nuevas variedades liberadas por AGROSAVIA entre 2012 y 2021 en 24 cultivos (Tabla 1); La lista de especies conservadas en el banco de germoplasma vegetal colombiano que incluye 275 cultivos y parientes silvestres (Reyes-Herrera, P. Pers. Comm, https://www.agrosavia.co/media/3542/lista_especies_bgv_2016.pdf); y la lista de cultivos subutilizados y marginales publicada previamente en diferentes trabajos de investigación de Colombia, la región andina y el mundo [4-6].

Tabla 1. Número de variedades de cultivo liberadas por AGROSAVIA e inscritas en la lista nacional de variedades (Registro Nacional de Cultivares, RNC - ICA) entre 2012 y 2021 en 24 cultivos. Cuatro variedades de las 69 fueron registradas en colaboración con otras instituciones: dos variedades de maíz con el CIMMYT, una variedad de frijol con Harvestplus y el CIAT, y una variedad de pasto con el CIAT. Además, siete de las 69 nuevas variedades utilizaron como material parental al menos una accesión conservada en el Banco de Germoplasma Vegetal de Colombia: dos de cebolla y cinco de papa (grupo Phureja).

	Año de liberación y registro

Nombre del cultivo	Nombre científico	2012	2013	2014	2015	2016	2017	2018	2019	2020	2021	Número total de variedades por cultivo
Arracacha	*Arracacia xanthorrhiza*	-	-	-	-	-	-	-	1	-	-	1
Frijol	*Phaseolus vulgaris*	-	-	-	2	-	-	-	-	-	-	2
Yuca	*Manihot esculenta*	-	-	-	-	2	8	1	-	-	-	11
Marañón	*Anacardium occidentale*	-	3	-	-	-	-	-	-	-	-	3
Cacao	*Theobroma cacao*	-	-	2	-	-	2	-	-	-	-	4
Maíz	*Zea mays*	1	1	1	-	-	1	-	3	-	-	7
Algodón	*Gossypium hirsutum*	1	2	-	-	-	-	3	-	-	-	6
Uchuva	*Physalis peruviana*	-	-	-	-	2	-	-	-	-	-	2
Guayaba	*Psidium guajava*	-	-	-	-	-	2	-	-	-	-	2
Pasto guinea	*Megathyrsus maximus*	-	-	-	-	1	-	-	-	1	-	2
Avena forrajera	*Avena sativa*	-	-	-	-	-	-	1	-	-	-	1
Palma de aceite	*Elaeis oleifera x Elaeis guineensis*	-	-	2	-	-	-	-	-	-	-	2
Cebolla	*Allium fistulosum*	-	-	-	-	2	-	-	-	-	-	2
Pasto brachiaria	*Brachiaria brizantha*	-	-	-	-	-	-	-	-	1	-	1
Ají dulce	*Capsicum chinense*	-	-	-	-	-	-	-	-	-	1	1
Papa	*Solanum tuberosum*	-	-	-	-	1	-	-	-	-	-	1
Papa criolla	*Solanum tuberosum* group Phureja	-	-	-	-	-	3	-	2	2	-	7
Arroz	*Oryza sativa*	-	-	-	-	-	-	1	-	-	-	1

Sorgo forrajero	*Sorghum bicolor*	-	1	-	-	-	-	-	-	-	-	1
Guanábana	*Annona muricata*	-	-	-	-	-	-	-	2	-	-	2
Soya	*Glycine max*	-	3	-	-	-	-	-	2	-	-	5
Caña de azúcar	*Saccharum officinarum*	-	-	-	1	1	-	-	-	-	-	2
Batata	*Ipomoea batatas*	-	-	-	-	-	-	2	-	-	-	2
Maní forrajero	*Arachis pintoi*	-	-	-	-	-	-	-	-	1	-	1
**Número total de variedades**		**2**	**10**	**5**	**3**	**9**	**16**	**8**	**10**	**5**	**1**	**69**

2.2.2. La conexión del PB&PGR con el banco de germoplasma nacional y con instituciones externas

Contamos el número de veces que los 52 investigadores utilizaron el Banco Nacional de Germoplasma en sus proyectos, así como el número y los nombres de todas las instituciones externas con las que consideran clave para futuros trabajos de colaboración.

2.2.3. La identificación de los puntos fuertes y débiles del programa de PB&PGR

Pedimos a los 52 investigadores que indicaran si estaban muy en desacuerdo, en desacuerdo, neutrales, de acuerdo o muy en desacuerdo con las siguientes diez afirmaciones asociadas a la identificación de los puntos fuertes y débiles en las diferentes actividades y competencias del área:

- El grupo de PB&PGR debería participar en el asesoramiento y la formación de estudiantes y jóvenes profesionales.
- El grupo de PB&PGR debería abrir espacios para los clubes de revistas y la discusión de la investigación.
- Los estudiantes y jóvenes profesionales tienen un buen conocimiento de la genética cuantitativa.
- Los estudiantes y jóvenes profesionales tienen un buen conocimiento del trabajo de campo.
- Los investigadores de PB&PGR tienen un buen conocimiento del MAS (Marker-Assisted Selection).
- Los investigadores de PB&PGR tienen un buen conocimiento del diseño experimental.
- Los investigadores de PB&PGR tienen un buen conocimiento de los modelos G (Genético) x E (Ambiente).
- Los investigadores de PB&PGR tienen un buen conocimiento de la genética cuantitativa.
- Los investigadores de PB&PGR tienen un buen conocimiento de la mejora genética molecular de las plantas .

- Los investigadores de PB&PGR tienen un buen conocimiento de la mejora vegetal participativa.

Por último, diseñamos una pregunta abierta en la que pedíamos comentarios o sugerencias para mejorar el grupo de PB&PGR en el futuro. Como obtuvimos 31 respuestas diferentes, realizamos un análisis de minería de textos del corpus que incluía un análisis de sentimientos y una nube de palabras. El primer paso fue traducir los 31 textos del español al inglés utilizando el software gratuito DEEPL (https://www.deepl.com/translator) y revisarlos a mano. A continuación, limpiamos el corpus utilizando la función R GSUB y el paquete R TM 0.7-7 para eliminar puntuaciones, números, espacios vacíos, palabras de parada y crear una lista de palabras asociadas al lugar de trabajo y al área de investigación (por ejemplo, AGROSAVIA, mejora vegetal, recursos fitogenéticos, etc.). Por último, utilizando el paquete R STRINGR 1.4.0, descompusimos cada texto en sus caracteres para compararlos con una lista de palabras positivas y otra negativa [24,25]. Tanto las palabras positivas como las negativas recibieron un peso de uno cada una. Así, generamos una puntuación de sentimiento para cada texto, restando el número de palabras negativas de las positivas. Si la puntuación era positiva, la opinión tenía un sentimiento positivo, y viceversa. Calculamos la media y el error estándar de las puntuaciones de sentimiento de las 31 opiniones. También generamos una nube de palabras basada en las 30 palabras más frecuentes del corpus utilizando el paquete R WORDCLOUD 2.6.

2.3. La construcción de una red social basada en los vínculos dentro del grupo PB&PGR y externos

En esta parte de la encuesta, dos investigadores no reportaron ninguna interacción interna o externa. Por lo tanto, construimos una red social bidireccional para el análisis, formada por 50/52 investigadores del grupo PB&PGR, que especificaba 812 interacciones dentro del grupo y 117 interacciones con investigadores externos (es decir, tanto con otras disciplinas dentro de AGROSAVIA como con instituciones externas).

Solicitamos reportar seis clases de interacciones entre investigadores, como la colaboración en proyectos en el área de PB&PGR, la discusión sobre nuevos avances en el área de PB&PGR, el intercambio o solicitud de germoplasma, la preparación de artículos científicos y literatura gris, el registro de nuevas variedades y el asesoramiento técnico. Comparamos el número de interacciones entre los investigadores del grupo del PB&PGR frente a los investigadores del PB&PGR y los investigadores externos para cada clase. Además, dentro de la red calculamos y clasificamos el grado y la influencia de cada investigador (es decir, vértice). El grado mide el número de interacciones adyacentes (es decir, aristas) con el paquete R IGRAPH 1.0.1 [26].

Comparamos las puntuaciones de los diez primeros grados con el grado académico más alto de los investigadores del PB&PGR para probar la hipótesis de que los investigadores con grados educativos más altos tendrán un mayor número de interacciones. Además, medimos la influencia de cada investigador basándonos en dos estadísticas relacionadas: las puntuaciones de centro y de autoridad. La puntuación del hub mide la capacidad de un investigador para relacionarse con otros investigadores influyentes (es decir, autoridades). Así, los investigadores

hub son aquellos que trabajan junto a investigadores de confianza en un tema común. En comparación, la puntuación de autoridad mide el número de enlaces de interacciones que un investigador obtuvo. Por lo tanto, los investigadores con autoridad deben tener información relevante sobre el campo y recibir más enlaces de otros investigadores. Estas dos puntuaciones se refuerzan mutuamente porque un buen hub apunta a muchas autoridades competentes, y una autoridad adecuada es señalada por muchos buenos hubs [27]. Asociamos el top diez de las puntuaciones de los hubs y de las autoridades con la información sobre el género de los investigadores del PB&PGR para probar la hipótesis de que la influencia de los investigadores es independiente del género.

También calculamos cuatro estadísticas descriptivas para la red (es decir, el ancho, la densidad de bordes, la distancia media y la transitividad) para diagnosticar cómo los investigadores trabajan juntos dentro y fuera del grupo PB&PGR. El diámetro, la densidad y la distancia media indican conjuntamente el grado de conexión de la red. En otras palabras, miden la capacidad de la información para atravesar el sistema. Así, para las redes con menos intermediarios a través de las interacciones y mayores conexiones directas, esperamos un diámetro menor, una densidad mayor y una distancia media menor. Asimismo, la transitividad mide la estructura a escala local de la red. Por tanto, una transitividad débil sugiere que las interacciones se producen en grupos poco conectados. En comparación, una transitividad alta indica un sistema bien consolidado sin posibilidad de identificar subgrupos internos discretos [28].

3. Resultados

3.1. La descripción del grupo

Encontramos una disparidad de género entre los investigadores que trabajan en PB&PGR dentro de AGROSAVIA, donde el 71% (n=37) son hombres, y el 29% (n=15) son mujeres, y ninguno declaró una identidad fuera del binario hombre-mujer. El 40% (n=21) de los investigadores hombres y mujeres tienen entre 31 y 40 años. Los investigadores hombres están presentes en todos los rangos de edad, pero las investigadoras mujeres son más frecuentes en los rangos de edad más jóvenes. Sólo dos investigadoras se sitúan en la franja de 51 a 70 años, y ninguna tiene más de 60 años (Figura 1A). La encuesta mostró que la proporción de investigadores con M.Sc. y los que tienen un doctorado es de 2:1. Nueve de los investigadores con M.Sc. se encuentran en la categoría de Asociado o Senior, ya que han alcanzado una productividad destacada. Además, los investigadores hombres están presentes en todos los rangos y edades de experiencia de AGROSAVIA dentro del grupo PB&PGR. En comparación, las investigadoras mujeres sólo se distribuyen en cinco de las ocho categorías, y tienen mayoritariamente entre 1 y 10 años de experiencia. Ninguna investigadora tiene más de 30 años de experiencia (Figura 1B). En lo que respecta a la formación, el 52% (n=27) son investigadores de maestría (20 hombres y siete mujeres) y el 27% (n=14) son investigadores de doctorado (siete hombres y siete mujeres) (Figura 1B). Al considerar sólo los grados académicos inferiores, tanto los hombres como las mujeres estudiaron sólo en Colombia. Sin embargo, existe un desequilibrio de género asociado a los estudios en el extranjero. De los 37 investigadores hombres, 14 (nueve

con maestría y cinco con doctorado) estudiaron en el extranjero. En cambio, de las 15 investigadoras, sólo cuatro obtuvieron su doctorado en el extranjero (Figura 1C).

3.2. Los retos y las oportunidades en el ámbito del PB&PGR

3.2.1. Lista de cultivos con interés de investigación y métodos de mejora utilizados

Los investigadores de AGROSAVIA tienen un amplio espectro de experiencia en investigación en al menos 31 cultivos, de los cuales 29 son originarios de los trópicos, siendo las únicas excepciones la soya y la mora. Además, de los 31 cultivos, 17 (55%) son considerados NUS (Tabla 2). Como resultado, el 40% (28 de 69) de las nuevas variedades liberadas por AGROSAVIA desde 2012 se encuentran en ocho de los 17 NUS (47%) (Tabla 2). Entre ellas se encuentran el frijol, el cacao, el maíz, la guayaba, el ají, la papa, la guanábana y la batata (Tabla 1).

Además, de esta lista de 31 especies, 19 (61%) mostraron un mayor interés en la investigación (es decir, más investigadores interesados en proyectos futuros que los que trabajan actualmente en ellos), tres especies mostraron igual importancia entre los planes de investigación pasados y futuros, y nueve especies mostraron un interés decreciente (es decir, pocos investigadores interesados en proyectos futuros que los que trabajan actualmente en ellos) (Figura 2). Por último, los investigadores han utilizado la selección clonal, la selección masiva y la selección asistida por marcadores con mayor frecuencia que otros métodos de mejora. Las nuevas metodologías de mejora, como la selección genómica y la transformación genética, se encuentran al final de la lista, con sólo un investigador con experiencia en cada categoría (Figura 3).

Tabla 2. Lista de cultivos en los que trabajan o han trabajado los investigadores de AGROSAVIA en los últimos diez años (ver Figura 2), y si están o no catalogados como especies desatendidas y subutilizadas (NUS) en tres niveles diferentes: Colombia, Andes y mundial [4-6]. Los cultivos están en orden alfabético mostrando el nombre científico cuando es aplicable (NA - no aplicable).

**Nombre del cultivo**	**Nombre científico**	**Listado como especie desatendida o subutilizada**		
		**Colombia**	**Andes**	**Mundo**
Aguacate	*Persea americana*	YES	NO	NO
Bananos y plátanos	*Musa spp.*	NO	NO	NO
Frijol	*Phaseolus vulgaris*	NO	YES	NO
Mora	*Rubus* spp.	NO	NO	YES
Yuca	*Manihot esculenta*	NO	YES	YES
Cítricos	*Citrus* spp.	NO	NO	YES
Cacao	*Theobroma cacao*	NO	YES	NO
Maíz	*Zea mays*	YES	NO	NO
Algodón	*Gossypium spp.*	NO	NO	NO
Berenjena	*Solanum melongena*	NO	NO	YES

Pastos	NA	NO	NO	NO
Uchuva	*Physalis peruviana*	NO	NO	NO
Guayaba	*Psidium guajava*	YES	YES	YES
Lulo/Naranjilla	*Solanum quitoense*	NO	NO	NO
Mango	*Mangifera indica*	NO	NO	NO
Palma de aceite	*Elaeis guineensis*	NO	NO	NO
Cebolla	*Allium cepa*	NO	NO	NO
Passifloras	*Passiflora spp.*	YES	YES	YES
Chontaduro	*Bactris gasipaes*	YES	NO	YES
Ají	*Capsicum baccatum*	NO	YES	YES
Papa	*Solanum tuberosum*	NO	YES	NO
Quinua	*Chenopodium quinoa*	YES	YES	YES
Arroz	*Oryza sativa*	NO	NO	NO
Caucho	*Hevea brasilensis*	NO	NO	NO
Sorgo	*Sorghum bicolor*	NO	NO	NO
Guanábana	*Annona muricata*	NO	YES	YES
Soya	*Glycine max*	NO	NO	NO
Caña de azúcar	*Saccharum officinarum*	NO	NO	NO
Batata	*Ipomoea batatas*	NO	YES	NO
Tomate de árbol	*Solanum betaceum*	YES	NO	YES
Tabaco	*Nicotiana tabacum*	NO	NO	NO

3.2.2. La conexión del PB&PGR con el banco nacional de germoplasma y las instituciones externas

Aunque el Banco Nacional de Germoplasma conserva 26 de las 31 especies (84%) de la lista de la Tabla 2, la encuesta mostró que este banco de germoplasma, también administrado por AGROSAVIA, no es el objetivo principal de los programas de premejoramiento y mejoramiento. El 37% (N=19) de los investigadores nunca ha utilizado el banco de germoplasma, y el 40% (N=21) lo ha utilizado sólo una o dos veces en los últimos diez años (Figura 4). Como resultado, sólo el 17% de las nuevas variedades liberadas desde 2012 tiene al menos un progenitor de una accesión conservada en el banco nacional de germoplasma (es decir, ocho nuevas variedades de 69 registradas) (Tabla 1).

Además, los investigadores que trabajan en el PB&PGR están interesados en mantener o establecer colaboraciones con 54 universidades e instituciones de investigación dentro y fuera de Colombia. La más frecuente fue la Universidad Nacional de Colombia, con 23 investigadores interesados en colaborar. Además, 29 investigadores quieren tener colaboraciones con tres de las bases del CGIAR (Grupo Consultivo para la Investigación Agrícola Internacional) dentro y fuera de Colombia, como el CIAT (Colombia), el CIMMYT (México) y el CIP (Perú). El CIAT y el CIMMYT se han asociado previamente con AGROSAVIA para liberar nuevas variedades de maíz,

frijol y pasto (Cuadro 1). Por último, Embrapa (Brasil), CIRAD (Francia) y Cenicaña (Colombia) contaron con al menos seis investigadores en busca de colaboraciones (Figura 5).

3.2.3. La identificación de los puntos fuertes y débiles del programa de PB&PGR

Descubrimos que los investigadores percibían debilidades en el trabajo de campo, en la selección asistida por marcadores (MAS), en el diseño experimental y en las habilidades de fitomejoramiento participativo. Además, los investigadores consideraban que debían asesorar y formar activamente a los estudiantes y jóvenes profesionales y liderar los clubes de revistas y los espacios de debate sobre investigación (Figura 6). La opinión de 31 de los 52 investigadores coincidió en tres estrategias principales para mejorar el programa de PB&PGR: (1) trabajar como una red en la que los diferentes investigadores del PB&PGR se beneficien de los conocimientos de otros miembros del grupo en varias áreas y disciplinas, (2) trabajar conjuntamente con otros investigadores que sean fuertes en otras materias como la fisiología vegetal y la fitopatología de las plantas, y (3) mejorar el impacto global centrándose en muy pocos cultivos estratégicos mientras se desarrolla un programa de investigación multidisciplinar a largo plazo. Las cinco palabras más utilizadas fueron "programas", "red", "conocimiento", "mejora" e "investigadores" (Figura 7). Las opiniones y las palabras más frecuentes encierran una puntuación de sentimiento positiva con una media de 1,55 (0,3 SE) en los 31 textos analizados.

3.3. La construcción de una red social basada en los vínculos dentro del grupo PB&PGR y externos

La red construida incluyó a 167 investigadores: 50 del grupo PB&PGR (con metadatos asociados) y 117 externos, bien de AGROSAVIA o de otras instituciones. Los 167 investigadores tuvieron 812 interacciones bidireccionales entre ellos. Encontramos diferencias en el número de interacciones según los tipos analizados. El registro de nuevas variedades tuvo el menor número de interacciones (N=50), mientras que la colaboración en proyectos de la zona tuvo el mayor número de interacciones (N=242) dentro de la red. Además, sólo dos tipos de vínculos entre investigadores (es decir, el intercambio de germoplasma o la solicitud y discusión sobre nuevos avances en el área) mostraron más interacciones dentro del grupo de PB&PGR que entre PB&PGR e investigadores externos (Figura 8).

En toda la red, el diámetro fue 8, la densidad de bordes fue 0,03 y la distancia media fue 3,07. Estos valores sugieren una red poco rígida con muchos investigadores intermedios a través de las interacciones entre los investigadores. Asimismo, la transitividad fue de 0,17. Este bajo valor indica un sistema débil caracterizado por varios subgrupos discretos e identificables.

En el centro de la red se encuentran los investigadores con más influencia (es decir, más interacciones con otros investigadores). Por el contrario, en la periferia se encuentran principalmente los investigadores externos (Figura 9A). Los diez investigadores con más interacciones (es decir, más grados en la red) son todos del grupo de PG&PGR, principalmente

hombres (N=8) con un grado de maestría (N=6) con 20 años de experiencia en el campo (N=7) que planean jubilarse durante los próximos dos años (Figura 9B).

Los diez investigadores más influyentes mostraron un rango más amplio de experiencia en el área e incluyeron a tres investigadores externos en la lista de autoridades. Sin embargo, al igual que en el caso de la titulación, son principalmente hombres (es decir, N=8 en ambos grupos), normalmente con un título de maestría (es decir, ocho y seis para los centros y las autoridades, respectivamente). A pesar de que las mujeres son una minoría como personas influyentes, una investigadora obtuvo la puntuación más alta tanto en los centros como en las autoridades (Figura 9C y 9D).

4. Discusión

4.1. Los antecedentes y las redes de colaboración

La creciente población mundial, los desafíos climáticos y la pérdida de agrobiodiversidad son los principales focos de atención de los fitomejoradores y los gestores de recursos fitogenéticos [1]. En este contexto, los cultivos tropicales, especialmente los categorizados como NUS, ofrecen una oportunidad que requiere que los investigadores trabajen juntos utilizando un enfoque transdisciplinario [4-6]. Como encontramos en nuestra encuesta, los investigadores de AGROSAVIA que trabajan en PB&PGR tienen un alto nivel de logros académicos y una amplia experiencia en mejoramiento vegetal, fisiología vegetal y producción de plantas, entre otros. Aunque esta experiencia es esencial para ayudar a desarrollar y avanzar en un programa de investigación eficaz en los próximos años, nuestro análisis apoyó la hipótesis de un personal del PB&PGR fragmentado que percibe un bajo impacto en su investigación. Estas brechas están asociadas al género, a los cambios generacionales y a las colaboraciones internas y externas. Por lo tanto, representan áreas en las que AGROSAVIA debería centrarse en mejorar en los próximos años para tener un mejor impacto en nuestras sociedades.

Nuestros resultados mostraron un fuerte sesgo de género en la composición de los investigadores que trabajan en mejora vegetal y recursos genéticos. Aunque la proporción total de mujeres y hombres en toda AGROSAVIA en 2017 fue de 0,613, solo fue de 0,408 en el grupo de PG&PGR encuestado para este estudio [29]. Varios estudios centrados en la igualdad de género muestran que las ciencias agrícolas son un campo dominado por los hombres [30,31]. Las disciplinas de las ciencias de los cultivos, la horticultura y la ingeniería agrícola tienen en todo el mundo una proporción entre mujeres y hombres de 0,435, 0,449 y 0,241, respectivamente [32]. Además, según la UNESCO, las mujeres que trabajan en las ciencias agrícolas y veterinarias sólo representan el 37,6% de la población investigadora [33]. Esta disparidad de género también es evidente en las ciencias agronómicas y veterinarias de Colombia, donde la proporción entre mujeres y hombres es de 0,769 para la licenciatura, 0,787 para la maestría y 0,694 para el doctorado [34]. Por otra parte, la Facultad de Agronomía de la Universidad Nacional de Colombia en Bogotá, que es la mayor universidad pública del país y una de las principales colaboradoras en el PB&PGR, tiene una relación mujer/hombre de 0,568 dentro de los

estudiantes y de 0,389 dentro de los profesores [35]. Por esta razón, aunque el profesorado representa sólo el 2,7% de la población total, es el tercero con mayor proporción de hombres entre todos los grupos de profesores de la universidad [36]. Por lo tanto, si no se toman medidas, la fuerza de trabajo en ciencias agrícolas en Colombia no alcanzará la paridad de género hasta dentro de aproximadamente 25 años [37].

Además, nuestros resultados mostraron que un menor número de investigadoras en AGROSAVIA obtuvieron sus títulos de maestría y doctorado en el extranjero comparado con los hombres. Aunque no teníamos ninguna pregunta en la encuesta que pudiera determinar directamente la causa, otros indicadores externos nos permitieron hipotetizar que las mujeres se enfrentan a más barreras sociales en Colombia para estudiar en el extranjero que los hombres. El cuidado y las tareas domésticas no remuneradas están fuertemente sesgadas hacia la población femenina en Colombia en base a dos indicadores: el número total (89,5% de las mujeres frente al 62% de los hombres que asumen esas responsabilidades) y el tiempo (7 horas diarias dedicadas a tareas no remuneradas para las mujeres frente a 3,5 horas para los hombres). Así, el 12,7% de las mujeres declaró no tener tiempo libre frente al 8,1% de los hombres [38]. Además, las científicas colombianas suelen empezar su doctorado más tarde que los hombres, y suelen tener ya responsabilidades de cuidado no remuneradas [39]. Por lo tanto, la combinación de cuidados no remunerados y tareas domésticas fuertemente sesgadas hacia las mujeres, un entorno académico patriarcal, y la segregación vertical y horizontal mientras se persigue una carrera científica pueden estar obstaculizando la movilidad de las investigadoras para estudiar en el extranjero [34,36]. Los futuros estudios sobre este tema en AGROSAVIA deben incluir un enfoque interseccional de género y desarrollo para comprobar esta hipótesis [40,41]. Esto debería incluir preguntas específicas sobre el sexo y la identidad de género, la edad para cursar estudios de posgrado, el tiempo dedicado a los cuidados no remunerados y a las tareas domésticas, y las experiencias directas de discriminación o las barreras por género, raza y clase social al solicitar estudios de posgrado en el extranjero o al inicio de sus carreras de investigación, entre otras.

Por otro lado, el análisis de la red nos permitió identificar una distribución desequilibrada entre los diez investigadores más influyentes en la PB&PGR por el grado más alto y el sexo [42]. Así, la red mostró una concentración de interacciones (es decir, puntuación de grado) basada principalmente en investigadores hombres con un grado de maestría y mucha experiencia en el área. Además, estos investigadores mostraron el mayor número de conexiones con otros actores externos, probablemente porque su trayectoria investigadora les ha permitido ampliar el número de colegas con los que interactúan. A pesar de ello, la comunidad investigadora aún no está bien consolidada. Tal y como habíamos previsto, la colaboración directa es escasa. Los subgrupos productivos están aislados y en la periferia de la red, generando clusters poco conectados con escasas colaboraciones fuera de estos subgrupos (Figura 8). Aunque AGROSAVIA debería centrarse en estos actores clave (nodos) como la mejor manera de comunicar y transferir la innovación dentro de toda la red PB&PGR, el principal obstáculo es que estos líderes identificados están cerca de su edad de jubilación en los próximos cuatro años. El análisis de la red también mostró que los investigadores de AGROSAVIA mantienen fuertes vínculos con muchos investigadores externos no incluidos en la encuesta. Este resultado sugiere

que esos actores extranjeros son consultores influyentes para el diseño de la estrategia de un programa de mejoramiento. Por lo tanto, los acuerdos de colaboración que AGROSAVIA está construyendo con varias instituciones dentro y fuera del país deben fomentar la formulación de proyectos para mantener y fortalecer estas colaboraciones externas conocidas.

Los datos recogidos en este estudio apoyan fuertemente la urgencia de tener una discusión interna sobre la mejora de la diversidad y la inclusión durante los próximos cambios generacionales dentro del personal del PB&PGR [34,37,39]. Actualmente, AGROSAVIA es una institución de investigación agronómica floreciente. Por lo tanto, promover esta discusión nacional internamente con una perspectiva de género y desarrollo daría a AGROSAVIA indicadores específicos de brechas que podrían ser monitoreadas a través del tiempo y corregidas con estrategias explícitas (ver este ejemplo para construir indicadores en conferencias [43]). Una política de apoyo a la diversidad y la inclusión también aumentaría la reputación de AGROSAVIA, convirtiéndola en un lugar de trabajo atractivo, especialmente para los investigadores del área agronómica que se identifican como mujeres y otras identidades de género u orientaciones sexuales no binarias. Además, es una oportunidad para asegurar la retención de todo el personal joven de investigación de doctorado [37,44]. Asimismo, esta discusión interna sobre las brechas y la inclusión podría ser una excelente oportunidad para promover el desarrollo agrícola con perspectiva de género en los diferentes proyectos liderados por AGROSAVIA [45,46].

4.2. Experiencia y competencias de investigación en la disciplina

Los investigadores que trabajan en fitomejoramiento y recursos fitogenéticos en AGROSAVIA tienen dos intereses principales en investigación actual y futura: 1) las frutas tropicales subutilizadas para los mercados internacionales y 2) la soberanía y seguridad alimentaria (Figura 2). Sin embargo, como ocurre en varios países, las actuales políticas colombianas para mejorar la seguridad alimentaria y desarrollar los mercados internacionales no identifican necesariamente a los NUS como una alternativa viable para lograr esos objetivos [47]. El maíz, por ejemplo, está categorizado como NUS en Colombia y es el primer cultivo de la lista de la Figura 2. Muchos investigadores están involucrados en el mejoramiento y la genética del maíz, siete nuevas variedades han sido liberadas desde 2012, y muchos más investigadores están interesados en trabajar con él en el futuro [45-51]. Este resultado coincide con informes anteriores, ya que Colombia consume 6,2 millones de toneladas de maíz al año. Además, este cultivo es de vital importancia para la seguridad alimentaria y la economía de los pequeños agricultores [45,52,53]. A pesar de ello, Colombia importa más del 80% de su maíz, principalmente de Estados Unidos y Argentina [54-57]. Adicionalmente, ningún programa público de mejoramiento de maíz se centra actualmente en las variedades de polinización abierta o en los híbridos para satisfacer las necesidades de los pequeños agricultores en Colombia y América Latina [58]. Aunque México se enfrentaba al mismo problema que Colombia en la actualidad, donde las importaciones ponían en peligro la producción local de maíz, encontraron una solución [48,49]. En 2010, el CIMMYT (Centro Internacional de Mejoramiento de Maíz y Trigo) y la SAGARPA (Secretaría de Agricultura, Ganadería, Desarrollo Rural, Pesca y Alimentación) del Gobierno mexicano, pusieron en marcha con éxito MasAgro. El

objetivo de esta iniciativa conjunta es aumentar la productividad, la rentabilidad y la sostenibilidad del maíz [59]. Los esfuerzos de MasAgro aumentaron significativamente la producción de maíz y la autosuficiencia alimentaria mediante la implementación de prácticas de gestión específicas, semillas híbridas y ventas directas en los mercados de maíz [60].

El diseño de un objetivo similar a largo plazo en Colombia para el maíz y otros NUS identificados en este estudio requiere sinergias entre la investigación pública del PB&PG y el gobierno colombiano que garanticen una comercialización efectiva independientemente de las tendencias del mercado global [4-6]. La experiencia previa en Europa sugiere que la financiación estatal es obligatoria para generar NUS competitivos y económicamente viables [50]. En este sentido, la investigación de PB&PG en AGROSAVIA debería centrarse en la clasificación de los distintos NUS con base a varios indicadores asociados a las tendencias del mercado y a la experiencia de investigación previa que identificamos en este estudio. A continuación, los investigadores del PB&PG deberían construir una hoja de ruta colaborativa utilizando fondos estatales para su investigación e incorporando nuevas tecnologías como la genómica y otros enfoques fenómicos para determinar el valor nutricional y la adaptabilidad de estos NUS a varios entornos con una perspectiva de cambio climático [47,51,52]. Además, esta investigación debería incluir enfoques de fitomejoramiento participativos, comunitarios y centrados en el género, en concertación con el gobierno [47,53-55].

Además, el éxito de los programas de fitomejoramiento de NUS suele comenzar con el uso de una amplia base de germoplasma genético. Por lo tanto, la recolección, la conservación y la caracterización de los recursos genéticos son obligatorias para la introgresión de nuevos alelos en los materiales de élite [52]. Colombia cuenta con un amplio Banco Nacional de Germoplasma Vegetal con una extensa colección de cultivos nativos e introducidos subutilizados [61]. Tres factores actuales están ayudando a mejorar esta situación en el futuro. En primer lugar, desde 2015, el Banco Nacional de Germoplasma Vegetal ha estado trabajando en un ambicioso proyecto de cinco años de duración destinado a implementar una plataforma GrinGlobal de fácil uso para curadores y usuarios [62]. En segundo lugar, como parte del mismo proyecto, se están empezando a utilizar nuevos enfoques, como la genómica, para una caracterización sólida de las colecciones [53]. En la actualidad, se han caracterizado dos colecciones de cultivos críticos, la papa [63] y el cacao [64], y están disponibles para el público. Pronto, esperamos publicar resultados similares para otros cultivos nativos e infrautilizados como el aguacate (*Persea americana*), *Passiflora* spp. y chontaduro (*Bactris gasipaes*). En tercer lugar, a partir de 2018, AGROSAVIA fue delegada oficialmente por el Ministerio de Agricultura para gestionar los Bancos de Germoplasma para la alimentación y la agricultura [17]. Por lo tanto, esperamos que una combinación de gestión activa, caracterización genómica, fenotipado de alto rendimiento y selección genómica aumente la precisión de la entrada de nuevo germoplasma en un programa de mejora [65,66].

4.3. Perspectivas de alianzas futuras

Este estudio también reveló las alianzas estratégicas básicas que AGROSAVIA está construyendo con instituciones nacionales y regionales centradas en cultivos convencionales (Figura 4). Por

ejemplo, Colombia está validando tecnologías y variedades generadas en otras instituciones, como la yuca, el frijol, los forrajes y el arroz del CIAT, el maíz del CIMMYT y la papa del CIP. Además, la nueva colaboración con instituciones de investigación y universidades de alto nivel, como la Universidad de Wageningen, el CIRAD y el USDA, abre nuevas posibilidades para codirigir iniciativas internacionales de investigación que utilicen tecnologías de vanguardia y enfoques de mejora genética novedosos, lo que facilitará el paso de ser adoptadores de tecnología a ser generadores de conocimiento y tecnología. Además del contexto actual de una economía basada en el conocimiento en la que la experiencia desempeña un papel vital en el crecimiento económico, la creciente relación con las universidades como actor fundamental en el sistema nacional de innovación se hace imprescindible. Un ejemplo de ello es Corea del Sur, que ha pasado de ser un estado en desarrollo a un país desarrollado [56]. Por ello, el trabajo sinérgico y colaborativo de AGROSAVIA con las universidades públicas y privadas debe ser una prioridad. Sin embargo, el modelo de innovación también implica a la industria en un paradigma de triple hélice en el que las universidades, la industria y el gobierno tratan de entender y cooperar entre estos componentes para impulsar el rendimiento de la innovación nacional [57].

Los investigadores encuestados expresaron situaciones preocupantes como la inclusión generacional, la impotencia, las capacidades restringidas y las escasas oportunidades de formación en nuevas áreas (Figuras 6 y 7). Nuestros resultados sugieren que los investigadores se enfrentan a un cambio influenciado por el nuevo modelo de trabajo en red y la entrada de al menos siete nuevos investigadores en el área de la genética y el fitomejoramiento en los últimos cuatro años. Esta situación puede ayudar a explicar las actitudes de resistencia individual que suelen estar regidas por la ansiedad que provocan los cambios repentinos de edades, experiencias, productividades científicas y capacidades del actual grupo de mejora vegetal en AGROSAVIA [58,59]. Aunque estos resultados requieren de análisis más detallados, la primera aproximación sugiere que AGROSAVIA necesita atender rápidamente este conjunto de preocupaciones para estructurar y consolidar un grupo robusto de PB&PGR, especialmente en las áreas de selección genómica y transformación genética.

El análisis de las redes de mejoramiento en maíz y trigo en México mostró patrones similares a los que encontramos en este estudio [60]. Por lo tanto, esta metodología de redes sociales podría extenderse en el futuro para que el grupo de mejoramiento vegetal maneje genes de resistencia en diferentes cultivos [61], para entender la dinámica de las distribuciones de semillas [62], y para determinar estrategias en la producción de granos [63]. Además de esto, los análisis adicionales utilizando las bases de datos de encuestas y publicaciones nacionales proporcionarán un amplio panorama de cómo los científicos de plantas en Colombia están colaborando con el fin de mejorar la gobernanza institucional y aumentar y apoyar estas relaciones de colaboración [64].

5. Conclusiones

Este estudio es fundamental para entender los retos y las oportunidades de los investigadores en PB&PGR en AGROSAVIA, una institución con financiación estatal que intenta retomar su

liderazgo tras sufrir diez años de falta de apoyo financiero público. A partir de nuestro análisis, proponemos cinco estrategias: 1) La promoción de la discusión interna sobre las brechas de género y los cambios generacionales con el fin de diseñar indicadores para monitorear y disminuir esta disparidad en el tiempo. 2) La construcción de iniciativas y sinergias de largo plazo con el gobierno colombiano para apoyar la producción local de cultivos de seguridad alimentaria independientemente de las tendencias del mercado. 3) Una mejor colaboración entre el Banco Nacional de Germoplasma Vegetal y los investigadores de fitomejoramiento. 4) Una lista concertada de especies prioritarias (especialmente de especies olvidadas y subutilizadas) e instituciones externas para enfocar mejor los esfuerzos de colaboración en la investigación de fitomejoramiento con fondos públicos. 5) Mejores espacios para la ideación y diseño de proyectos entre los investigadores, así como programas de capacitación en nuevas tecnologías asociadas al mejoramiento genético de plantas y al manejo de recursos fitogenéticos. Además, sugerimos crear un grupo consultivo que incorpore estos cinco principios en las propuestas de investigación de alto impacto para generar nuevos cultivares que respondan a los retos bióticos y abióticos de la agricultura nacional.

Materiales complementarios: Apéndice A: Preguntas de la encuesta en español e inglés, Apéndice B: Todas las respuestas de la encuesta organizadas por secciones en un archivo Excel, Apéndice C: El manuscrito en español.

Contribuciones de los autores: Conceptualización, C.H.G., H.J. y C.G.; metodología, C.H.G. y K.T.; análisis formal, C.H.G., K.T. e I.C.S.; redacción-borrador original, C.H.G. e I.C.S.; redacción-revisión y edición, C.H.G. e I.C.; Todos los autores han leído y aceptado la versión publicada del manuscrito.

Financiación: Esta investigación fue financiada por el Ministerio de Agricultura y Desarrollo Rural de Colombia.

Declaración de la Junta de Revisión Institucional: Este estudio se realizó de acuerdo con las directrices del comité de bioética de AGROSAVIA.

Declaración de consentimiento informado: Se obtuvo el consentimiento informado de todos los sujetos involucrados en el estudio durante el segundo taller de fitomejoramiento y recursos fitogenéticos celebrado del 22 al 24 de marzo de 2017.

Declaración de disponibilidad de datos: Todos los datos generados en este estudio están disponibles en la información complementaria cambiando los nombres y la información personal por un número de investigador.

Agradecimientos: Queremos agradecer a todos los investigadores del PB&PGR que accedieron voluntariamente a llenar la encuesta y discutieron activamente los resultados durante el segundo taller de mejoramiento vegetal y recursos fitogenéticos celebrado del 22 al 24 de marzo de 2017. Gracias a Jhon Freddy Hernández por el diseño de la encuesta google y el análisis piloto de los datos obtenidos. Gracias a los compañeros William Burgos, Andrés J.

Cortés, Jhon Jacobo Cañas, Víctor Nuñez, Martín Rodríguez, José Vicente Romero y Edison Julián Ramírez por liderar el comité para el diseño de una política de mejoramiento a largo plazo para AGROSAVIA. Gracias a la Dirección Técnica de Semillas (ICA) y al Departamento de Propiedad Intelectual de AGROSAVIA por facilitar la información sobre las variedades de cultivo liberadas por AGROSAVIA entre 2012 y 2021. Gracias a los dos revisores anónimos que nos ayudaron a mejorar este manuscrito. Por último, deseamos extender nuestro especial agradecimiento a la Dra. Kali M. Brandt, que revisó el estilo en inglés de la versión final.

Conflictos de intereses: Los autores declaran no tener ningún conflicto de intereses.

Apéndice A
Las preguntas de la encuesta en español e inglés.

Apéndice B
Todas las respuestas de la encuesta organizadas por secciones en un archivo Excel.

Apéndice C
El manuscrito en español.
