# Supplementary material for: Opportunities and Challenges to Improve a Public Research Program in Plant Breeding and Enhance Underutilized Plant Genetic Resources in the Tropics
Source: Genes (Basel). 2021 Oct 9;12(10):1584. doi: 10.3390/genes12101584 (PMC8535561; doi:10.3390/genes12101584)
Supplement: Supplementary file 1 [file genes-12-01584-s001.zip › Supplementary Materials Table S1.pdf]

Appendix 1. The survey questions of the study, presented in Spanish (original) and English (translation). The initials PB&PGR means plant breeding and plant genetic resources.

**Español**	**English**
**Descripción del grupo**	**Group description**
Nombre	Name
Género (femenino, masculino, otro)	Gender (male, female, other)
Rango de edad (20-30, 31-40, 41-50, 51-60, 61-70)	Age range (20-30, 31-40, 41-50, 51-60, 61-70)
Último estudio terminado (pregrado, especialización, maestría, doctorado)	Highest academic degree (bachelor's degree, graduate certificate degree, M.Sc., Ph.D.)
Escalafón dentro de AGROSAVIA (profesional de apoyo, profesional de apoyo a la investigación, consultor, Investigador master, Investigador master asociado, investigador master senior, Investigador PhD, investigador PhD asociado)	AGROSAVIA's rank (research professional, research support professional, consultant, M.Sc. Researcher, M.Sc. Associate researcher, M.Sc. Senior researcher, PhD.D. Researcher, Ph.D. Associate researcher)
¿Su último estudio lo realizó fuera de Colombia? (si, no)	Did you last study is abroad? (yes, no)
**Retos y oportunidades en el area PB&PGR**	**Challenges and opportunities in the PB&PGR area**
Por favor mencione los cultivos en que usted ha desarrollado actividades de fitomejoramiento dentro de los últimos 10 años	Please mention the crops in which you have developed breeding activities within the last 10 years
Mencione los cultivos más importantes en los que AGROSAVIA debe trabajar en mejoramiento genético a futuro	Please mention the most important crops in which AGROSAVIA should work on genetic improvement in the future
Por favor mencione los métodos o sistemas de mejoramiento que ha aplicado en sus programas de mejoramiento en los últimos 10 años	Please mention the breeding methods you have applied in your breeding programs in the last 10 years
¿Cuántas veces ha solicitado accesiones al banco de germoplasma de la nación en los últimos 10 años?	How many times have you requested accessions from the nation's germplasm bank in the last 10 years?
El grupo de investigadores PB&PGR debería participar en la formación y entrenamiento de estudiantes y nuevos profesionales (Totalmente en desacuerdo, en desacuerdo, neutro, de acuerdo, totalmente de acuerdo)	The PB&PGR group should participate in advising and training students and young professionals (Strongly disagree, disagree, neutral, agree, strongly agree)
El grupo de investigadores PB&PGR debería propiciar espacios abiertos para clubes de revistas y discusión en investigación (Totalmente en desacuerdo, en desacuerdo, neutro, de acuerdo, totalmente de acuerdo)	The PB&PGR group should open spaces for journal clubs and research discussion (Strongly disagree, disagree, neutral, agree, strongly agree)

Los estudiantes y profesionales jóvenes tienen un buen conocimiento de genética cuantitativa (Totalmente en desacuerdo, en desacuerdo, neutro, de acuerdo, totalmente de acuerdo)	Students and young professionals have a good knowledge of quantitative genetics (Strongly disagree, disagree, neutral, agree, strongly agree)
Los estudiantes y profesionales jóvenes tienen un buen conocimiento del trabajo de campo (Totalmente en desacuerdo, en desacuerdo, neutro, de acuerdo, totalmente de acuerdo)	Students and young professionals have a good understanding of fieldwork (Strongly disagree, disagree, neutral, agree, strongly agree)
Los investigadores PB&PGR tienen un buen conocimiento en selección asistida por marcadores (Totalmente en desacuerdo, en desacuerdo, neutro, de acuerdo, totalmente de acuerdo)	PB&PGR researchers have a good understanding of MAS (Marker-Assisted Selection) (Strongly disagree, disagree, neutral, agree, strongly agree)
Los investigadores PB&PGR tienen un buen conocimiento en diseño de experimentos (Totalmente en desacuerdo, en desacuerdo, neutro, de acuerdo, totalmente de acuerdo)	PB&PGR researchers have a good knowledge of experimental design (Strongly disagree, disagree, neutral, agree, strongly agree)
Los investigadores PB&PGR tienen un buen conocimiento en modelos de interacción genotipo x ambiente (Totalmente en desacuerdo, en desacuerdo, neutro, de acuerdo, totalmente de acuerdo)	PB&PGR researchers have a good understanding of G (Genetic) x E (Environment) models (Strongly disagree, disagree, neutral, agree, strongly agree)
Los investigadores PB&PGR tienen un buen conocimiento en genética cuantitativa? (Totalmente en desacuerdo, en desacuerdo, neutro, de acuerdo, totalmente de acuerdo)	PB&PGR researchers have a good understanding of quantitative genetics (Strongly disagree, disagree, neutral, agree, strongly agree)
Los investigadores PB&PGR tienen un buen conocimiento en mejoramiento molecular (Totalmente en desacuerdo, en desacuerdo, neutro, de acuerdo, totalmente de acuerdo)	PB&PGR researchers have a good knowledge of molecular plant breeding (Strongly disagree, disagree, neutral, agree, strongly agree)
Los investigadores PB&PGR tienen un buen conocimiento en mejoramiento genético participativo (Totalmente en desacuerdo, en desacuerdo, neutro, de acuerdo, totalmente de acuerdo)	PB&PGR researchers have a good understanding of participatory plant breeding (Strongly disagree, disagree, neutral, agree, strongly agree)
Si tiene comentarios o sugerencias para mejorar la configuración del grupo PG&PGR por favor escribalos en esta sección.	If you have comments or suggestions to improve the configuration of the PG&PGR group please write them in this section
**Conexiones dentro del grupo PB&PGR y externos**	**Links within the PB&PGR group and externals**
Por favor mencione los investigadores dentro de la corporación con los que usted más frecuentemente ha ejecutado sus proyectos en	Please mention the researchers within the corporation with whom you have most frequently executed your projects in genetics and plant breeding in the last 10 years

genética y fitomejoramieto en los últimos 10 años	
Por favor mencione los investigadores dentro de la corporación con los que usted más frecuentemente ha consultado o pedido asesoría técnica en sus investigaciones en los últimos 10 años	Please list the researchers within the corporation with whom you have most frequently consulted or sought technical advice on your research in the last 10 years
Por favor mencione los investigadores dentro de la corporación con los que usted más frecuentemente ha escrito sus artículos científicos o material divulgativo en los últimos 10 años	Please list the researchers within the corporation with whom you have most frequently written your scientific articles or dissemination material in the last 10 years
Por favor mencione los investigadores dentro de la corporación con los que usted más frecuentemente ha registrado sus nuevas variedades en los últimos 10 años	Please list the researchers within the corporation with whom you have most frequently registered your new varieties in the last 10 years
Por favor mencione los investigadores dentro de la corporación con los que usted más frecuentemente ha discutido los mas recientes avances en el area del fitomejoramiento y la genética vegetal en los últimos 10 años	Please list the researchers within the corporation with whom you have most frequently discussed the most recent advances in plant breeding and plant genetics in the last 10 years
Por favor mencione los investigadores dentro de la corporación con los que usted más frecuentemente ha intercambiado y/o solicitado germoplasma de sus programas de mejoramiento en los últimos 10 años	Please list the researchers within the corporation with whom you have most frequently exchanged and/or requested germplasm from your breeding programs in the last 10 years
Mencione las instituciones fuera de AGROSAVIA con quienes a usted le gustaría y/o le parece estratégico trabajar en el futuro cercano en fitomejoramiento y genética	Please mention the institutions outside AGROSAVIA with whom you would like and/or think it is strategic to work with in the near future in plant breeding and genetics
